# Artery of Percheron infarction: review of literature with a case report

**DOI:** 10.2478/raon-2014-0037

**Published:** 2015-03-25

**Authors:** Urska Lamot, Ivana Ribaric, Katarina Surlan Popovic

**Affiliations:** 1 Clinical Institute of Radiology, University Medical Centre Ljubljana, Ljubljana, Slovenia; 2 Department of Vascular Neurology and Intensive Therapy, Neurology Clinic, University Medical Centre Ljubljana, Ljubljana, Slovenia

**Keywords:** Percheron, infarction, imaging

## Abstract

**Background.:**

Clinical features indicating an ischemic infarction in the territory of posterior cerebral circulation require a comprehensive radiologic examination, which is best achieved by a multi-modality imaging approach (computed tomography [CT], CT-perfusion, computed tomography angiography [CTA], magnetic resonance imaging [MRI] and diffusion weighted imaging [DWI]). The diagnosis of an acute ischemic infarction, where the damage of brain tissue may still be reversible, enables selection of appropriate treatment and contributes to a more favourable outcome. For these reasons it is essential to recognize common neurovascular variants in the territory of the posterior cerebral circulation, one of which is the artery of Percheron.

**Case report.:**

A 69 year-old woman, last seen awake 10 hours earlier, presented with two typical clinical features of the artery of Percheron infarction, which were vertical gaze palsy and coma. Brain CT and CTA of neck and intracranial arteries upon arrival were interpreted as normal. A new brain CT scan performed 24 hours later revealed hypodensity in the medial parts of thalami. Other imaging modalities were not performed, due to the presumption that the window for the application of effective therapy was over. The diagnosis of an artery of Percheron infarction was therefore made retrospectively with the re-examination of the CTA of neck and intracranial arteries.

**Conclusions.:**

A multi-modality imaging approach is necessary in every patient with suspicion of the posterior circulation infarction immediately after the onset of symptoms, especially in cases where primary imaging modalities are unremarkable and clinical features are severe, where follow-up examinations are indicated.

## Introduction

The thalami and midbrain have a complex blood supply with a large number of feeding arteries.^1^,^2^ The arterial supply is provided by perforating branches from the posterior cerebral artery and the posterior communicating artery.^3^ Although there are significant variations and overlaps, the thalamic vascular supply is classically categorized into 4 territories: anterior, paramedian, inferolateral and posterior.^4^–^6^ In addition to the paramedian thalami, the paramedian thalamic arteries supply the medial areas of the upper brainstem: the interpeduncular nucleus, the decussation of the superior cerebellar peduncles, the medial part of the red nucleus, the third and fourth cranial nerve nuclei and the anterior portion of the periaqueductual grey matter.^3^,^7^

Consequently, occlusion of the artery of Percheron causes a bilateral paramedian thalamic infarction with or without midbrain infarction.^2^,^4^,^5^ Additional involvement of the anterior thalamus is uncommon.^5^ The prevalence of arteries of Percheron is unknown. Since strokes in these territories are infrequently diagnosed, it is not known whether they are really rare or highly underdiagnosed.^1^

Due to a large number of blood supply variants of the posterior cerebral circulation, an ischemic infarction in this territory presents variable and unspecific clinical symptoms, which requires a comprehensive radiologic examination. This approach enables a diagnosis of an ischemic infarction in the early stage, when treatment with thrombolysis and/or mechanical recanalization is still possible and therefore reversibly damaged brain tissue can be salvaged with prompt revascularization. The goal of this paper is to report a case where the diagnosis of an artery of Percheron infarction was made retrospectively, due to an unspecific clinical presentation and a deficient multi-modality approach.

### Clinical presentation

The complex anatomy and function of the human thalamus and its variable vascular supply are responsible for the extremely variable clinical features when this structure is damaged by an ischemic infarction; in addition, the vascular overlap with the underlying midbrain extends the spectrum of these clinical features to include midbrain signs.^5^,^9^ An ischemic stroke in the territory of an artery of Percheron usually presents with three main symptoms, which are found in patients with bilateral paramedian thalamic strokes. These are vertical gaze palsy (65%), memory impairment (58%) and coma (42%).^4^,^6^ Bilateral paramedian thalamic lesions are often accompanied by rostral midbrain lesions, producing a “mesencephalothalamic” or “thalamopeduncular” syndrome.^6^,^10^ In addition to the mentioned triad, the syndrome is characterized by other oculomotor disturbances, hemiplegia, cerebellar ataxia and movement disorders.^6^

### Imaging and treatment

Diagnosing an artery of Percheron infarction is critical for directing the appropriate time sensitive management and preventing additional unnecessary procedures.^6^,^11^ Different treatment methods, such as intravenous thrombolysis and endovascular treatment are available. They can be performed if a diagnosis of acute stroke is made.^4^ As in our case, the diagnosis is often made in the late stage, when therapy is ineffective and dangerous. Magnetic resonance imaging (MRI) usually allows visualization of the initial infarct in cases of acute cerebral ischemia and is used in stroke centres as the primary or early secondary imaging modality.^4^

Infarction of the artery of Percheron presents as an abnormal signal intensity on MRI and/or hypoattenuation on CT, involving the bilateral paramedian thalami with or without rostral midbrain involvement.^6^ Early diagnosis is best made by a diffusion weighted imaging (DWI) sequence using MRI.^11^

Lazzaro *et al*., identified four patterns of ischemic infarctions when the Percheron artery is occluded.^6^ Approximately 43% of their patients demonstrated damage to both paramedian thalami and midbrain, while 38% had ischemic damage to paramedian thalami only, without midbrain involvement. In around 14% of patients, the damage involved the anterior thalamic nuclei in addition to paramedian thalami and upper midbrain. The least common pattern (5%) was ischemic damage of the bilateral paramedian and anterior thalami; the midbrain was spared in these cases. They also found that a previously unreported finding (a “V” sign) on fluid attenuated inversion recovery (FLAIR) and DWI sequences was identified in 67% of cases of artery of Percheron infarction with midbrain involvement and this sign supports the diagnosis when present.^5^,^6^ The “V” sign appears as a distinct pattern of V-shaped hyperintensity on axial FLAIR and/or DWI along the pial surface of the midbrain adjacent to the interpeduncular fossa.^6^

The artery of Percheron is rarely visualized with conventional angiography and, to the best of our knowledge, only four authors have successfully demonstrated this variant^5^,^6^; it is too small to be visualized by computed tomography angiography (CTA) or magnetic resonance angiography (MRA).^11^

## Case presentation

A 68 year-old Caucasian woman, last seen awake 10 hours earlier, was found unresponsive in front of her apartment. The patient had a history of hypertension, but, it is unclear if she had used any long-term medication. On physical examination in our Neurological Emergency Room, she was comatose with Glasgow Coma Scale 4 and the following initial vital signs: pulse 60 beats/min, respiratory rate 16 breaths/min and blood pressure 110/53 mmHg. Pupillary light reflex in the right eye was non-reactive and the left eye poorly reactive. The right pupil was dilatated. Passive examination of the ocular movements showed complete vertical gaze palsy. The patient was afebrile and no meningeal signs were present. She flexed the right arm and extended the left leg on painful stimulus. Babinski could not be provoked on the left and was in flexion on the right. No other pathological signs were present. The following laboratory tests were all unremarkable: complete blood cell count, glycaemia, electrolytes, liver enzymes, creatinine, ammonia, arterial blood gas values, ethanol, benzodiazepine and opioid levels. The international normalized ratio (INR) was 1.15. In this case the neurological examination was misleading due to unspecific clinical signs and symptoms which were unhelpful in the process of achieving the correct working diagnosis.

**FIGURE 1.**
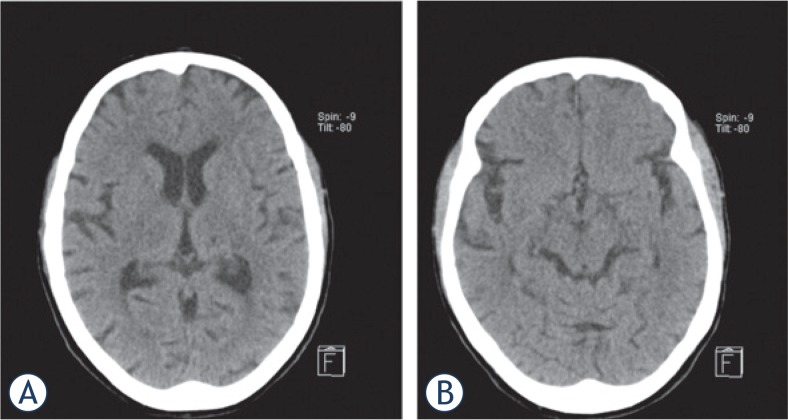
The non-contrast head CT scan performed on the day of admission was normal, in particular without early signs of ischemia at the level of both thalami **(A)** the mesencephalon **(B)**.

**FIGURE 2.**
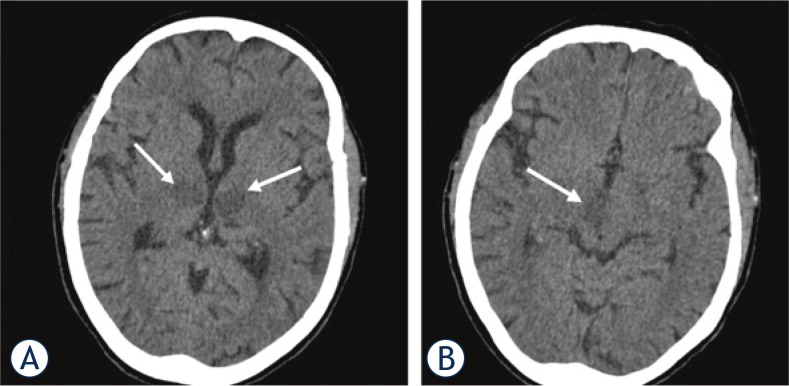
Another non-contrast head CT scan was performed 24 hours later and showed symmetrical ill-defined areas of hypodensity in the medial part of both thalami, corresponding to occlusion of the artery of Percheron (white arrows) **(A)**. The hypodense area in the right thalamus extended into the anterior part of the mesencephalon and cerebral peduncle. The hypodense area in the left thalamus extended only into the anterior part of the mesencephalon (white arrow) **(B)**.

The initial CT performed 60 minutes after finding the patient (and an unknown time after loss of consciousness) showed no acute haemorrhage or early signs of ischemia, only an old lacunar infarction in the left thalamus (Figure 1). CTA of neck and intracranial arteries was interpreted as normal. A new head CT was performed 24 hours later. It revealed areas of hypodensity (16× 10 mm) in the medial thalami, which were not present on the previous CT examination (Figure 2). The right hypodense area extended into the anterior part of the mesencephalon and cerebral peduncle. The left hypodense area extended only into the anterior part of the mesencephalon. The chronic ischemic change in the posterior part of the left thalamus remained unchanged.

**FIGURE 3.**
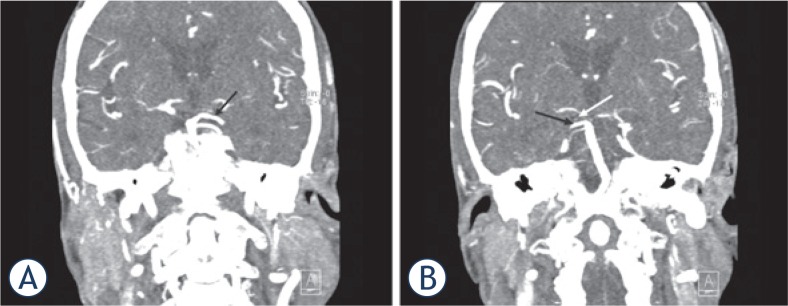
Computed tomography angiography (CTA) of the neck and intracranial arteries. The basilar artery and the left posterior cerebral artery (PCA) (black arrow) were both transient **(A)**. At first glance, the right PCA appears to be fully opacified. Detailed examination of the CTA images revealed a filling defect of the P1 segment of the right PCA (white arrow) and another rare anatomic variant: duplication of the right superior cerebellar artery (black arrow), which could have been mistaken for a transient right PCA **(B)**.

Due to our findings on the second CT, we decided to perform another reading of the CTA examination performed at the time of the patients’ admission, which revealed a duplication of the right superior cerebellar artery and a filling defect of the P1 segment of the right posterior cerebral artery (Figure 3).

There was no improvement in the patients’ neurological status in the following days and she was included in our palliative care program. On day 5, the patient became febrile with raised inflammatory parameters. Although we applied an antibiotic, the patient’s clinical status deteriorated and she died of cardiopulmonary failure on the 13^th^ day of hospitalization. This study was conducted according to the principles of the Declaration of Helsinki and approved by the institutional ethical committee.

## Discussion

The large number of variants of the blood supply in the posterior cranial fossa, especially the high variability of presence and size of P1 segments, which give rise to the paramedian arteries, may be a clue that the artery of Percheron is not such an infrequent variant and may be underdiagnosed.^1^,^6^ The characteristic artery of Percheron infarct pattern has been estimated in different studies to occur in 0.1 to 2%^5^,^7^ of all ischemic strokes and in 4% to 18% of all thalamic strokes.^6^

**FIGURE 4.**
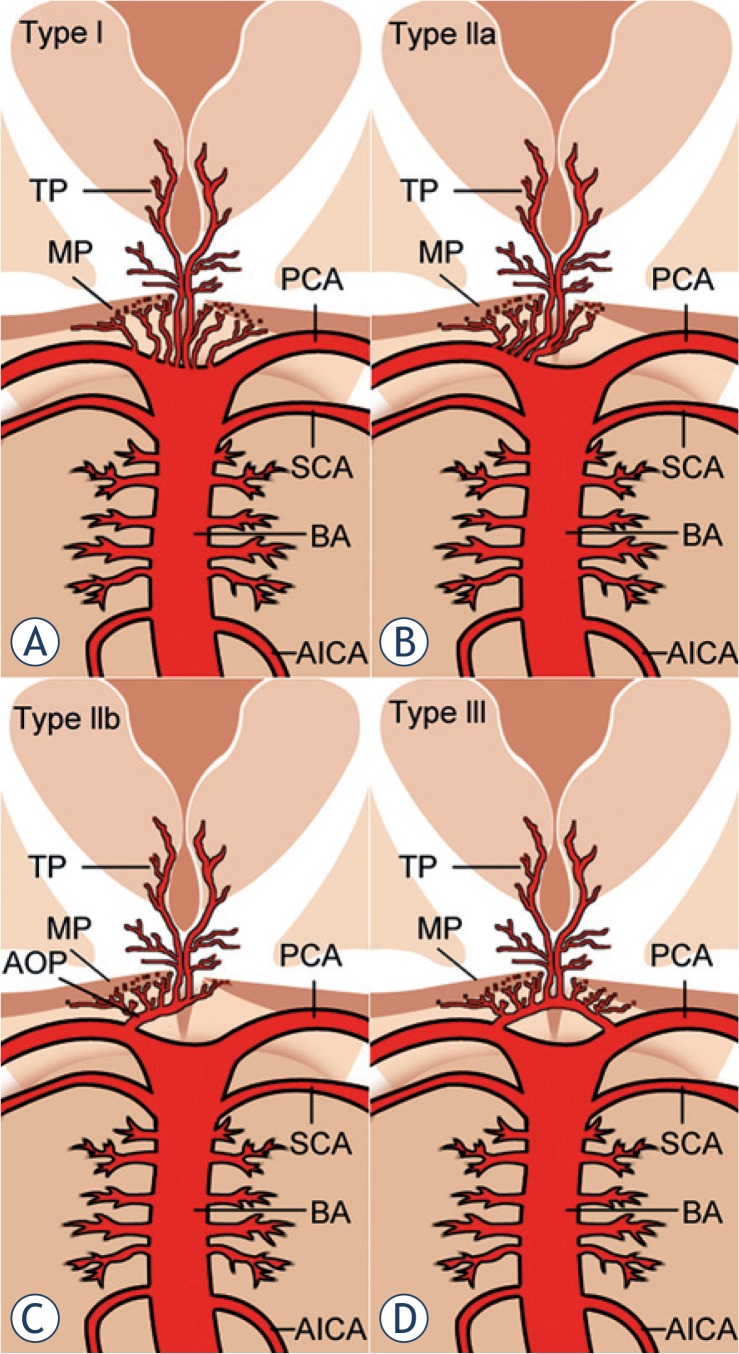
Anatomic variations of the arterial supply to the paramedian thalamic-mesencephalic region as described by Percheron: Variant I **(A)**, variant IIa **(B)**, variant IIb **(C)** – the artery of Percheron, variant III **(D)**. Vessels marked by initials: thalamic perforators (TP), midbrain perforators (MP), posterior cerebral artery (PCA), superior cerebellar artery (SCA), basilar artery (BA), anterior inferior cerebellar artery (AICA) and artery of Percheron (AOP).

According to Percheron, there are four normal variants of the neurovascular anatomy of the thalami and midbrain.^4^,^8^ Variant I is most common, in which each perforating artery arises from each left and right posterior cerebral artery (Figure 4A).^4^,^5^ Variant IIa is a less common, asymmetrical variant, in which perforating arteries arise directly from the proximal segment of one of the posterior cerebral arteries (Figure 4B).^3^–^5^ In variant IIb, the bilateral perforating thalamic arteries arise from a single arterial trunk called the artery of Percheron, which arises from the P1 segment of one posterior cerebral artery. It supplies the paramedian thalami and the rostral midbrain bilaterally (Figure 4C).^2^–^5^ Variant III is an arcade variant, with several small perforating branches arising from a single arterial arc that bridges the P1 segments of both posterior cerebral arteries (Figure 4D).^3^,^5^

It is difficult to suspect bithalamic paramedian infarcts because of the complex anatomy, which causes large clinical variability.^4^ They are typically characterized by a triad of altered mental status, vertical gaze palsy and memory impairment.^6^ Our patient presented with two of the three typical features of this stroke syndrome; that is, vertical gaze palsy and altered mental status.

A head CT was performed on admission to exclude haemorrhage, tumours, other obvious brain lesions and early signs of brain ischemia. A reexamination of the CTA revealed an overlooked anatomical variant, a duplication of the right superior cerebellar artery and a filling defect of the P1 segment of the right posterior cerebral artery. In our previous reading, we had mistaken one of the duplicated right superior cerebellar arteries for a transient P1 segment of the right posterior cerebral artery. Due to the aforementioned findings, we assume that right and left paramedian thalamic arteries aroused from a common trunk, since the P1 segment of the left posterior cerebral artery was transient. A falsely negative CTA of neck and intracranial arteries omitted the consideration of mechanical revascularization, for the possibly effected vessels were not visualized. In such cases where clinical findings are severe we suggest prompt further examination using other imaging modalities. On the basis of clinical, neuroimaging and neurovascular findings, the only possible diagnosis was an ischemic stroke in the territory of the artery of Percheron and this diagnosis was therefore made retrospectively. These findings demonstrate that when an artery of Percheron is suspected, the possibility that other rare anatomic variants of the posterior circulation may be present should also be considered.

In a patient with an acute onset of a neurological deficit and changes in the described locations, a diagnosis of a stroke of an artery of Percheron must be considered.^1^ The prognosis of artery of Percheron infarction may be ameliorated by treatment of acute stroke. Patients with acute ischemic stroke are thrombolysed intravenously (application of alteplase) unless there are contraindications. Endovascular revascularization applies thrombolytic agents directly into the thrombus or mechanically extracts the cloth.^12^ Mechanical trombectomy is considered in patients with a diagnosis of acute stroke, who have an occlusion of a treatable intracranial artery and are within 10.5 hours of onset of posterior circulation symptoms, to allow recanalization within 12 hours.^4^,^12^ The most applicable sites for interventional exploration are carotid T occlusion, M1 and M2 segments of the medial cerebral artery occlusion and vertebra-basilar thrombosis.^12^ As in our case, the diagnosis is often made many hours or even days after the clinical onset. At this stage therapy is ineffective and dangerous. Endovascular treatment is only seldom an option in these cases, for these arteries are often too small for visualization during the procedure.

MRI normally allows visualization of the initial infarct in cases of acute cerebral ischemia and is usually used in stroke centres as the primary or early secondary imaging modality. When brain MRI shows an acute stroke, thrombolysis can be performed if the deadline for achieving it is not over.^4^ Early diagnosis is best made using a DWI sequence.^1^ As in other locations in the brain, the combination of pathologic DWI and normal findings on T2-weighted or FLAIR images suggest an acute stroke. If the lesions are already visible on T2 or FLAIR images, the time window for thrombolysis is over.^1^,^4^ In our case, early MRI and CT perfusion were not performed because, on the basis of the patients’ medical history, we presumed the window for thrombolysis was over. Considering our findings, we should perhaps have performed MRI or CT perfusion, since they could have helped in the decision-making process.

To the best of our knowledge, there is only a single report in the literature of a symptomatic patient presenting an acute Percheron stroke with normal early brain MRI.^4^ On the basis of this one case, Cassourret^4^ *et al.* concluded that a normal initial MRI cannot formally eliminate the diagnosis of acute stroke of the artery of Percheron, although they state that the MRI was of inferior quality because the technical conditions were not optimal. The article suggests that, on the presentation of acute rostral brain stem stroke, accompanied by an inconclusive brain MRI, new brain imaging by MRI should be performed within therapeutic times or interventional explorations focused on the vertebrobasilar territory^4^ should be considered.

To the best of our knowledge, the value of CT perfusion in acute Percheron stroke has not been evaluated. The advantage of CT perfusion is that it is able to delineate areas of the brain that may be salvaged by intervention (e.g., thrombolysis or clot retrieval), known as the penumbra, from the parts that are irrevocably destined to go into infarct regardless of therapy, known as the infarct core. What is more, identification of infracted areas is easier than with non-contrast head CT. The weakness of CT perfusion at the level of the midbrain is that we often come across artefacts that reduce the quality of the examination.

It must be born in mind that the artery of Percheron is rarely visualized with conventional angiography^6^, since these vessels are too small^1^, and to our knowledge only 4 authors have successfully demonstrated this variant.^6^ Performing conventional angiography may not be indicated, because lack of visualization of the artery does not exclude its presence (because it is occluded).^2^ Although in our case it is unlikely that we would have visualized the artery of Percheron, we should perhaps have performed conventional angiography, since it could have shown the filling defect of the P1 segment of the right posterior cerebral artery, which would have influenced our decision regarding treatment. Conventional cerebral angiography should therefore not be used routinely to diagnose Percheron artery occlusion.^5^

When an ischemic infarction in the territory of the posterior circulation is suspected we firstly perform a native head CT to exclude haemorrhage and enable treatment with intravenous thrombolysis. In the case of negative imaging findings we continue the examination by performing a brain CT-perfusion and CTA of neck and intracranial arteries for the detection of ischemic infarctions not visible on native CT. When regardless the severe clinical picture all the mentioned imaging methods fail to depict the causative pathology for the deterioration of the patient’s state, we suggest performing MRI with T1-, T2- and FLAIR sequences and DWI for an exclusion of ischemic infarction in early stages.

We estimate the time to perform a comprehensive multi-modality examination in the evaluation of posterior circulation ischemic infarctions to 45 to 90 minutes, depending on the time from the insult to imaging, size of infarction and technical difficulties, for patients with posterior circulation infarcts usually require mechanical life support devices which make examination with MRI difficult.

The imaging differential of bithalamic lesions is broad and includes arterial and venous occlusion, infiltrative neoplasm, infectious and inflammatory lesions, and a single large embolus at the basilar tip could result in a similar infarct pattern. However, this would typically manifest as the “top of the basilar” syndrome with additional characteristic posterior circulation infarcts.^6^ The final diagnosis is based on the combination of clinical picture, laboratory tests and imaging findings.

Considering all the given information, we suggest that in emergency settings, in which the severity of the clinical features (coma and vertical gaze palsy) does not correlate with the imaging findings, the possibility of an artery of Percheron infarction must be taken into consideration and CT perfusion or MRI performed within therapeutic times, in order to make the correct diagnosis when treatment is still possible.
